# Effects and mechanism of moderate aerobic exercise on impaired fasting glucose improvement

**DOI:** 10.1186/s12944-015-0117-z

**Published:** 2015-12-02

**Authors:** Huo-cheng Liao, Si-gan Zhong, Peng Li, Wei-bin Chen, Cheng Cheng, Yue-gang Wang, Ping-sheng Wu, Chun Xiao

**Affiliations:** Department of Cardiology, the 3rd People’s Hospital, Huizhou, 516000 China; Department of Internal Medicine, Guangzhou Hospital, Guangzhou, 510080 China

**Keywords:** Moderate aerobic exercise, Rho-associated kinase, Impaired fasting glucose

## Abstract

**Background:**

Exercise is beneficial for blood glucose metabolism. However, whether moderate aerobic exercise could improve impaired fasting glucose is unknown. And the mechanism is also needed to investigate.

**Methods:**

A cross-sectional research was performed and 120 participants with impaired fasting glucose (IFG) were randomly assigned into active and controlled groups. Briefly, participants in active group were required to take moderate aerobic exercise at least 30 min for five times per week, whereas in controlled group, participants were also advised to take exercise but not mandatorily required the same degree as that of active group. At baseline and 3 month’s follow-up, laboratory and demographic variables were compared.

**Results:**

At baseline, no significant between-group differences were observed. Generally, leukocyte ROCK2 activity in the active and controlled groups were 58.7 ± 6.0 mg/mL and 60.2 ± 7.3 mg/mL, and daily average exercise time at baseline in both groups was extremely little, with 5.2 ± 3.8 min and 5.9 ± 3.5 min, respectively. After 3 months’ follow-up, 52 and 56 participants in the active and controlled groups completed the whole program. Compared to baseline, leukocyte ROCK2 activity and daily average exercise time were improved in both groups. Nonetheless, compared to the controlled group, leukocyte ROCK2 activity was reduced more profoundly and the daily average exercise time was longer in the active group (37.5 ± 6.3 min versus 18.3 ± 7.2 min, *p* < 0.05). Moreover, the percentage of IFG in the active group was decreased more prominently than the controlled group (76.9 % versus 82.1 %, *p* < 0.05). Multivariate regression analyses revealed that exercise time and leukocyte ROCK2 activity was significantly associated with IFG, with OR of 0.836 (active group versus controlled group, 95 % CI 0.825-0.852, *p* < 0.05) in exercise time, and 1.043 (controlled group versus active group, 95 % CI 1.021–1.069, *p* < 0.05) in leukocyte ROCK2 activity. In addition, exercise time was significantly associated with leukocyte ROCK2 activity, with OR of 0.822 (active group versus controlled group, 95 % CI 0.818–0.843, p < 0.05).

**Conclusion:**

In subjects with IFG, increased daily average exercise time is beneficial for improving fasting blood glucose metabolism, and the mechanism may be associated with its effects on attenuating leukocyte ROCK2 activity.

## Introduction

Currently, diabetes mellitus (DM) has become one of the most important risk factors for cardiovascular disease in both developed and developing countries [1]. Previously, some studies revealed that improving glucose metabolism by medicines could reduce adverse outcomes in subjects with coronary artery diseases [2, 3]. In addition, emerging evidence reveals that compared to the controlled participants, the incidences of ASCVD (mainly including ischemic stroke, coronary artery disease and peripheral artery disease) and DM in populations with pre-diabetic status such as impaired fasting glucose (IFG) are also profoundly increased [4–6]. Therefore, effectively and efficiently treating this subgroup of pre-diabetic population is clinical relevance to reduce the incident DM and ASCVD. Knowingly, glucose metabolism disorder contributes to the development and progression of atherosclerosis in patients with DM. For example, increased serum glucose level leads to endothelial dysfunction and thereby causes leukocytes infiltration and accumulation in vascular wall [7, 8]. In addition, hyperglycemia contributes to increased platelet activity which is positively associated with atherosclerotic plaque formation and progression [9].

ROCKs are important regulators of cellular apoptosis, proliferation, metabolism, and migration via control of the actin cytoskeletal assembly [10]. In the past decades, ROCKs have been identified associated with endothelial dysfunction, platelet activation, inflammatory reaction and oxidative stress, macrophages proliferation and endothelial apoptosis, and smooth muscle cells contraction in experimental researches [10–14]. Moreover, increased ROCK activity contributes to atherosclerosis progression and inhibiting ROCK activity by its inhibitor fasudil is beneficial for improving symptoms and clinical outcomes in patients with ASCVD [15, 16]. Knowingly, increased ROCK activity is associated with metabolic syndrome development [17] and lifestyle modification such as moderate aerobic exercise is helpful to improve metabolic disorder [18]. However, whether moderate aerobic exercise is beneficial for attenuating ROCK activity needs further investigation. Thus this, we conducted a cross-sectional clinical research to address these questions. Hopefully, results from our preliminary research could provide evidence and valuable data for future researches.

## Methods

### Participant enrollment

Totally, 120 participants who were diagnosed as IFG according to the criterion of guideline recommendation were enrolled [1]. All participants were without previous myocardial infarction, ischemic stroke, peripheral artery disease, severe liver and renal dysfunction, and anemia. Oral informed consent was obtained before enrollment. At baseline, all enrolled participants were randomly assigned into the active group and controlled group. Participants in the active group were recommended to mandatorily take at least 30 min of moderate aerobic exercise (jogging or brisk walking) at least 5 days per week. Briefly, watch or the time facility in the mobile-phone was used to record the duration of moderate aerobic exercise which was recorded on a paper. Similarly, participants in the controlled group were also advised to take exercise but were not mandatorily required to take the same degree of exercise as that in the active group. In terms of mandatory requirement, which meant that members of our research group called participants in the active group every 3 days by telephone to check out and encourage them to follow protocol’s instructions. However, participants in the controlled group would not get such kind of motivated encouragement. After 3 months’ follow-up, parameters of interest collected and compared between these two groups.

### Blood parameters measurement

At baseline and 3 months’ follow-up, fasting blood samples were drawn for the measurements of serological parameters including lipid profiles, liver and renal functions, high-sensitivity C-reactive protein (Hs-CRP), fasting blood glucose (FBG), fasting C-peptide and insulin levels, and glycated hemoglobin level (HbA1c). All the measurements were conducted in the laboratory center of our hospital.

### Demographic data collection

Demographic data including age, gender, smoking status, body mass index (BMI), waist circumference, systolic and diastolic blood pressure (SBP and DBP), heart rate, hypertension and dyslipidemia, daily average exercise time, current medicine usage were collected by two working staffs.

### Cytoplasm level of ROCK2 activity assessment

Fasting venous blood was drawn and leukocyte was isolated. The cytoplasm of leukocyte was obtained and stored at −80 °C until use. Cytoplasm level of ROCK2 activity was assessed by enzyme-linked immune-sorbent assay (ELISA kit, abcam company, ab160505). Briefly, at baseline and 3 months’ follow-up, fasting blood samples were drawn and centrifuged at 1200 g for 5 min, and then the cytoplasm of leukocyte was obtained and stored at −80 °C until use. All the procedures were according to the manufactures’ instruction. Optical density of each sample was detected by enzyme-labeling measuring instrument at a wavelength of 460 nm, and the activity was figured out on the standard curve. All the measurements were repeated for 3 times to figure out the arithmetic average.

### Statistical analysis

Continuous data was presented as mean ± SD or median (inter-quartile range) as appropriate, and was compared by the Student’s *t*-test when data was normally distributed, otherwise was compared by the Wilcoxon rank-sum test. Categorical data was presented as percentage and was compared by *χ*^2^ test. Multivariate regression analysis was conducted to evaluate the relationship between exercise and other variables with the ROCK2 activity and IFG incidence, respectively. All reported *p* values were 2-sided, and a *p* value of < 0.05 was considered statistically significant. All statistical analyses were conducted with the SPSS statistical package for Windows version 19.0 (SPSS Inc., Chicago, Illinois).

## Results

### Baseline characteristics of the active and controlled groups

Baseline characteristics of the active and controlled groups were evaluated and compared, and no significant between-group differences of these variables were observed (Table 1). Generally, male participant is predominant in present research. The BMI were 24.6 ± 3.3 kg/m^2^ and 24.8 ± 3.7 kg/m^2^, respectively, and nearly 31.7 % and 33.3 % of participants were overweight and obese. The percentages of participants with smoking (38.3 % versus 35.0 %), hypertension (26.7 % versus 30.0 %), and dyslipidemia (31.7 % versus 30.0 %) were comparable. Serum level of FBG, HbA1c and other serological indices were also no significant different. Serum levels of Hs-CRP were comparably increased in the active and controlled groups. Leukocyte cytoplasm ROCK2 activity in the active and controlled groups were 58.7 ± 6.0 mg/mL and 60.2 ± 7.3 mg/mL, respectively. Fasting C-peptide and insulin levels in both groups were below the normal lower limit and without significant difference between groups. Daily average exercise time at baseline in both groups was extremely little, with 5.2 ± 3.8 min and 5.9 ± 3.5 min, respectively. Current medicines usage for anti-platelet, hypertension and dyslipidemia were similar in both groups, and no medicine used for hyperglycemia was reported.

**Table 1:** Baseline characteristics of the active and controlled groups

Variables	Active (*n* = 60)	Controlled (*n* = 60)
Age (years)	42.4 ± 5.8	44.1 ± 6.6
Male, n (%)	33 (55.0)	35 (58.3)
BMI (kg/m^2^)	24.6 ± 3.3	24.8 ± 3.7
Overweight, n (%)	10 (16.7)	12 (20.0)
Obese, n (%)	9 (15.0)	8 (13.3)
Waist circumference (cm)	84.4 ± 7.6	82.9 ± 8.3
Smoking, n (%)	23 (38.3)	21 (35.0)
Hypertension, n (%)	16 (26.7)	18 (30.0)
Dyslipidemia, n (%)	19 (31.7)	18 (30.0)
SBP (mmHg)	127.8 ± 16.4	125.9 ± 15.7
DBP (mmHg)	79.8 ± 10.6	78.5 ± 11.7
HR (bpm)	73.7 ± 10.4	76.5 ± 9.9
TG (mmol/L)	1.8 ± 0.4	1.9 ± 0.3
TC (mmol/L)	5.7 ± 0.6	5.8 ± 0.5
LDL-C (mmol/L)	3.6 ± 0.3	3.7 ± 0.4
HDL-C (mmol/L)	1.1 ± 0.3	1.1 ± 0.2
Lp(a) (mg/L)	132.4 ± 15.6	135.7 ± 17.3
FBG (mmol/L)	6.3 ± 0.5	6.4 ± 0.4
HbA1c (%)	5.6 ± 0.4	5.6 ± 0.5
Cr (μmol/L)	98.7 ± 10.6	99.2 ± 9.9
BUN (mmol/L)	5.6 ± 1.3	5.9 ± 1.4
Hs-CRP (mg/L)	5.2 ± 1.1	5.6 ± 1.0
ROCK2 activity (mg/mL)	58.7 ± 6.0	60.2 ± 7.3
Fasting C-peptide level (nmol/L)	0.26 ± 0.05	0.28 ± 0.07
Fasting insulin level (pmol/L)	13.6 ± 2.2	14.1 ± 2.8
Daily average exercise time (min)	5.2 ± 3.8	5.9 ± 3.5
Aspirin, n (%)	28 (46.7)	26 (43.3)
ACEI, n (%)	9 (15.0)	7 (11.7)
ARB, n (%)	4 (6.7)	5 (8.3)
CCB, n (%)	4 (6.7)	3 (5.0)
Statins, n (%)	14 (23.3)	13 (21.7)

*BMI* body mass index, *SBP* systolic blood pressure, *DBP* diastolic blood pressure, *HR* heart rate (beat per minute), *TG* triglyceride, *TC* total cholesterol, *LDL-C* low-density lipoprotein cholesterol, *HDL-C* high-density lipoprotein cholesterol, *Lp(a)* lipoprotein (a), *FBG* fasting blood glucose, *Cr* creatinine, *BUN* blood urea nitrogen, *ACEI* angiotensin converting enzyme inhibitor, *ARB* angiotensin receptor blocker, *CCB* calcium channel blocker

### Variable comparison between groups after 3 months’ follow-up

After 3 months’ follow-up, six participants in the active group were dropout due to be unable to complete pre-set exercise program and two participants moved to other cities, and four participants in the controlled group were dropout due to move to other cities. Variables between groups were compared. As presented in Table 2, importantly, variables including BMI, waist circumference, and the percentages of obese and smoking were all comparably reduced in both groups compared to baseline (*p* < 0.05 for all comparisons). In addition, compared to baseline, the serological indices including lipid profiles, FBG, Hs-CRP, fasting C-peptide and insulin levels, and ROCK2 activity were improved in both groups. Nonetheless, after 3 months’ follow-up, compared to the controlled group, the percentage of overweight, serum levels of Lp(a) and Hs-CRP, and ROCK2 activity were reduced more profoundly in the active group (*p* < 0.05), and the increments of fasting C-peptide and insulin levels were more prominent in the active group (*p* < 0.05). The percentages of medicine usage including anti-platelet, anti-hypertension and statins were similarly increased in both groups. Notably, the daily average exercise time in both groups were significantly increased compared to baseline. However, compared to the controlled group, the exercise time in the active group was significantly longer (37.5 ± 6.3 min versus 18.3 ± 7.2 min, *p* < 0.05). Briefly, 48 participants (92.3 %) in the active group and seven participants (12.5 %) in the controlled group completed the exercise program during these 3 months follow-up. Moreover, the percentage of IFG in the active group was also significantly decreased than that in the controlled group (76.9 % versus 82.1 %, *p* < 0.05).

**Table 2:** Variable comparison between groups 3 months’ follow-up

Variables	Active (*n* = 52)	Controlled (*n* = 56)
Age (years)	43.7 ± 5.2	44.9 ± 6.0
Male, n (%)	28 (53.8)	31 (55.4)
BMI (kg/m^2^)	23.1 ± 3.5	24.2 ± 4.6
Overweight, n (%)*	5 (9.6)	10 (17.9)
Obese, n (%)	5 (9.6)	5 (8.9)
Waist circumference (cm)	81.2 ± 5.2	82.2 ± 7.6
Smoking, n (%)	18 (34.6)	18 (32.1)
Hypertension, n (%)	13 (25.0)	13 (23.2)
Dyslipidemia, n (%)	15 (28.8)	16 (28.6)
SBP (mmHg)	120.5 ± 11.7	122.4 ± 11.3
DBP (mmHg)	75.4 ± 7.8	75.1 ± 9.0
HR (bpm)	70.3 ± 8.3	73.1 ± 7.6
TG (mmol/L)	1.6 ± 0.3	1.8 ± 0.3
TC (mmol/L)	5.1 ± 0.5	5.5 ± 0.4
LDL-C (mmol/L)	3.2 ± 0.3	3.4 ± 0.4
HDL-C (mmol/L)	1.2 ± 0.2	1.1 ± 0.3
Lp(a) (mg/L)*	109.6 ± 10.7	121.3 ± 12.4
FBG (mmol/L)	6.0 ± 0.3	6.3 ± 0.5
HbA1c (%)	5.4 ± 0.4	5.6 ± 0.3
Cr (μmol/L)	95.4 ± 10.3	99.7 ± 9.2
BUN (mmol/L)	5.7 ± 1.1	5.9 ± 1.0
Hs-CRP (mg/L)*	3.4 ± 0.7	4.9 ± 1.1
ROCK2 activity (mg/mL)*	40.6 ± 5.3	53.3 ± 7.0
Fasting C-peptide level (nmol/L)*	0.89 ± 0.11	0.46 ± 0.10
Fasting insulin level (pmol/L)*	24.3 ± 4.6	19.7 ± 3.0
Aspirin, n (%)	29 (55.8)	30 (53.7)
ACEI, n (%)	10 (19.2)	9 (16.1)
ARB, n (%)	4 (7.7)	6 (10.7)
CCB, n (%)	5 (9.6)	4 (7.1)
Statins, n (%)	16 (30.8)	17 (30.4)
IFG, n (%)*	40 (76.9)	46 (82.1)

*p < 0.05

### Relationship between exercise and other variables with IFG

Multivariate regression analyses were performed to evaluate the relationship between exercise and other variables with IFG. After adjusting for age, gender, BMI, smoking, LDL-C and Lp(a), only Hs-CRP, exercise time, fasting C-peptide level, FBG, ROCK2 activity and statins were significantly associated with IFG. After additional adjusting for Hs-CRP, fasting C-peptide level and statins, merely exercise time and ROCK2 activity remained significantly associated with IFG, with odd ratio (OR) of 0.836 (active group versus controlled group, 95 % CI 0.825–0.852, *p* < 0.05) in exercise time, and 1.043 (controlled group versus active group, 95 % CI 1.021–1.069, *p* < 0.05) in ROCK2 activity.

### Relationship between exercise and other variables with ROCK2 activity

Multivariate regression analyses were performed to evaluate the relationship between exercise and other variables with ROCK2 activity. After adjusting for age, gender, BMI, smoking, LDL-C and Lp(a), only Hs-CRP, exercise time, FBG, and statins were significantly associated with ROCK2 activity. After additional adjusting for Hs-CRP and FBG, only exercise time remained significantly associated with ROCK2 activity, with OR of 0.822 (active group versus controlled group, 95 % CI 0.818–0.843, *p* < 0.05).

## Discussion

Diabetes mellitus imposes great economic and medical burden worldwide, and preventing the pre-diabetics progressing into overt DM is beneficial to reduce the incidence of DM and its associated complications such as ASCVD. Data from our preliminary research indicates that in subjects with IFG, 3 months of moderate aerobic exercise is beneficial for improving fasting glucose metabolism. In addition, other key variables including serum level of Hs-CRP and ROCK2 activity were also significantly decreased. Multivariate regression analyses revealed that exercise time and ROCK2 activity were independently associated with IFG. Moreover, exercise time was inversely associated with ROCK2 activity. These data suggested that increased moderate aerobic exercise was beneficial for improving IFG, and the mechanism might be associated with its effects on decreasing ROCK2 activity.

Accordingly, in subjects with IFG, the risk to develop overt DM is profoundly higher than those without IFG. Mechanically, in subjects with IFG, basic insulin production is significantly reduced and thereby leads to fasting blood glucose elevation. Increased serum glucose level is detrimental to vascular wall and could result in systemic inflammation and oxidation [7, 19]. As reflected in our present research, serum level of Hs-CRP at baseline was profoundly increased which is consistent with previous reports [19, 20]. However, whether increased ROCK2 activity is associated with the incidence of IFG, and in addition whether exercise is useful for decreasing ROCK2 activity has not been fully investigated yet.

Briefly, ROCK2 is an isoform of rho-associated kinase and is expressed in multiple tissues such as vascular wall and myocardium. Previously, many experimental studies have revealed that increased ROCK2 activity is associated with pathogenesis of cardiovascular diseases. Inhibiting ROCK2 activity by its inhibitor or cardiac-specific deletion of ROCK2 leads to profound cardio-protective effects including reduced cardiac fibrosis and diminished lipid accumulation in vascular wall [21, 22]. However, whether ROCK2 activity is associated with glucose metabolism is unknown yet. Our present cross-sectional research revealed that in subjects with IFG, 3 months of moderate aerobic exercise not only reduced the incidence of IFG, but also attenuated ROCK2 activity. Results from multivariate regression analyses indicated that through diminishing ROCK2 activity, exercise time improved fasting blood glucose metabolism in subjects with IFG. On the basis of pathophysiological functions of ROCK2, we considered that the following explanations might account for our findings. In the first place, it has been reported that increased ROCK activity is associated with phosphorylation of insulin receptor substrate-1 (IRS-1) which thereby causes insulin resistance and blood glucose elevation [23]. Secondly, since adipocytes are the key organ in keeping glucose homeostasis, and increased ROCK activity promotes inflammation and oxidation within adipocytes which thereby negatively affects glucose metabolism [24]. Indeed, in animal model, using ROCK inhibitor could result in improvement of glucose metabolism [25]. Last but not the least, increased ROCK activity is associated with BMI, waist circumference, and Hs-CRP level [26]. This suggests that increased ROCK activity could serve as a critical contributor for DM and metabolic syndrome development.

Another important finding of our present research was that exercise time is negatively associated with ROCK2 activity. On the basis of the physiological roles of exercise, we considered that the following mechanisms could be used to explain this relationship. On the first hand, exercise is beneficial for improving endothelial function and reducing oxidative stress [27], both of which were beneficial for attenuating ROCK2 activity. On the other hand, since ROCK activation is dependent on small GTP-binding protein Rho isoprenylation during cholesterol biosynthesis. Therefore, cholesterol metabolism improved by exercise might diminish Rho isoprenylation and its associated ROCK activation. Indeed, lipid profiles in both groups were improved after 3 months’ follow-up in our present research, and the benefits presented in an exercise-time dependent fashion.

Other than ROCK2 activity and IFG, other variables including lipid profiles, fasting C-peptide and insulin, and Hs-CRP levels were all improved in both groups compared to the baseline. And these benefits associated with the duration of daily average exercise time. Notably, although data from our present research indicates a relationship between exercise time and IFG, and the underlying mechanism may be associated with exercise effects on attenuating ROCK2 activity. However, we could not draw a causal relationship owing to the inherent defect of cross-sectional research. Rather, data from our present research could be used as a tool for hypothesis generation for future research.

Lastly, in our present research, we used the American Diabetes Association criterion to define IFG (plasma glucose levels 5.6–6.9 mmol/L) which is different from that of World Health Organization (WHO, 6.1 mmol/L as the cut-off value). Therefore, it is cautious to interpret our finding when using different diagnostic criterion. Additionally, the cut-off value to diagnose IFG in our present research was 5.6–6.9 mmol/L, therefore, we considered that finding regarding the positive relationship between ROCK2 activity and IFG might be applied to wider range of population than using WHO criterion. Moreover, since it was reported that IFG was predominantly related to genetic factors, while impaired glucose tolerance (IGT) was predominantly related to physical inactivity [28]. Therefore, it was possible that moderate aerobic exercise might also improve IGT in participants with IFG and ROCK2 activity might also be significantly associated with IGT which deserved further investigation.

## Conclusion

Our present research shows that in subjects with IFG, increased daily average exercise time is beneficial for improving fasting blood glucose metabolism, and the mechanism operating in this process may be associated with its effects on attenuating ROCK2 activity.
